# Transcutaneous Spinal Cord Stimulation Improves Upper and Lower Limbs’ Motor and Sensory Function in a Subject with Central Cord Syndrome: A Case Report

**DOI:** 10.3390/neurolint18020031

**Published:** 2026-02-10

**Authors:** Fernando Reyes, Camila Parker, Tania Turquie, Aldo Chimal, Lorermy Villalobos, Frida Bailey, Antonio Ibarra, Igor Lavrov, Carlos A. Cuellar

**Affiliations:** 1 Faculty of Health Sciences, Universidad Anáhuac México, Huixquilucan 52786, Mexico; fernando.reyesar@anahuac.mx (F.R.); camila.parkerin@anahuac.mx (C.P.); tania.turquieba@anahuac.mx (T.T.); frida.bailey@anahuac.mx (F.B.); 2 Becario de la Dirección General de Calidad y Educación en Salud, Secretaría de Salud, Mexico City 11570, Mexico; 3 Facultad de Medicina, Universidad Autónoma del Estado de México, Toluca 50000, Mexico; achimalm002@alumno.uaemex.mx (A.C.); lvillalobosb001@alumno.uaemex.mx (L.V.); 4 Centro de Investigación en Ciencias de la Salud (CICSA), Faculty of Health Sciences, Universidad Anáhuac México, Huixquilucan 52786, Mexico; jose.ibarra@anahuac.mx; 5 Neurology Department, Radiology Department, Mayo Clinic, Rochester, MN 55905, USA; lavrov.igorl@mayo.edu; 6 School of Sport Sciences, Universidad Anáhuac México, Huixquilucan 52786, Mexico

**Keywords:** central canal syndrome, spinal cord injury, transcutaneous spinal cord stimulation, physical therapy, spinal cord stimulation, non-invasive neuromodulation, trans-spinal stimulation

## Abstract

**Background:** Central cord syndrome (CCS) is the most common incomplete spinal cord injury, producing more severe motor deficits in the upper than lower extremities and impairing sensory and autonomic function. Although transcutaneous spinal cord stimulation (tSCS) has shown benefits in motor and sensory recovery after spinal cord injury, studies have not explicitly documented whether CCS subjects were included. The aim of this study was to assess the effects of tSCS over 12 weeks on motor and sensory outcomes in a subject with CCS. **Methods:** A 20-year-old male with a C7 injury was evaluated at baseline and after 12 weeks with the American Spinal Cord Injury Impairment scale, Modified Ashworth Scale, Penn and Spasm Frequency Scale, 3-Meter Walk Test and 6-Minute Walk Test, 9-Hole Peg Test, Box and Block Test, hand dynamometry, and lower-limb EMG. tSCS was applied between T9 and L1 at 30 Hz. **Results:** At 12 weeks, upper-limb motor and sensory scores improved, while spasm frequency and hand spasticity were reduced. Manual dexterity improved bilaterally in the 9-Hole Peg and Box and Block Tests, with a 2 kg gain in right-hand grip strength. In the 6-Minute Walk Test, the distance covered increased from 224.4 m to 295.2 m, and a 1.36 s reduction in 3-Meter walking time was achieved. **Conclusions:** tSCS improved motor and sensory function and reduced spasticity and spasms. These findings suggest that tSCS may serve as an effective complementary intervention for motor and sensory rehabilitation in individuals with mild cervical injuries, including CCS.

## 1. Introduction

The central cord syndrome (CCS) accounts for approximately 30% [1] to 70% [2] of all cases of incomplete tetraplegia in mixed-age cohorts. In particular, in young people, it represents about 20% of incomplete spinal cord injuries (SCIs), with high-energy trauma being the most common cause [3]. CCS is typically characterized by disproportionately greater motor impairment in the upper than in the lower extremities, frequently accompanied by deficits in fine motor control, impairments in postural stability and balance, disturbances in somatosensory perception, sphincter dysfunction, and neuropathic symptoms such as paresthesia, burning pain, or diffuse discomfort, depending on lesion extent and severity [4,5]. A retrospective study found a significant weakness in the distal upper limbs in 36% and in the lower limbs in 41% of the examined cohort [6].

Although CCS has been associated with a relatively favorable prognosis, current longitudinal analyses indicate that neurological and functional recovery tend to plateau within the first one to two years post-injury, often leaving persistent distal motor and sensory deficits that significantly influence autonomy, participation, and overall quality of life [7]. These residual impairments contribute to increased dependency and impose substantial demands on rehabilitative services, thereby emphasizing the need for therapeutic strategies that enhance functional restoration [8,9].

In recent years, transcutaneous spinal cord stimulation (tSCS) has emerged as a non-invasive neuromodulation approach to augment motor, sensory, and autonomic recovery following SCI [10,11,12,13,14,15,16]. This technique involves the placement of surface electrodes over the skin to deliver electrical currents that activate large-diameter afferent fibers within the dorsal roots, thereby modulating segmental and suprasegmental spinal circuits [17,18]. Multiple investigations have demonstrated that tSCS can enhance spinal excitability, facilitate the re-engagement of dormant or partially preserved pathways, and potentiate voluntary motor output, even in subjects diagnosed with motor-complete or sensory-complete spinal cord injury [19].

Although case series and case reports have included individuals classified as D according to the American Spinal Cord Injury Impairment scale (AIS), the literature has not clarified whether these studies included subjects with CCS, a distinction that is clinically relevant due to the unique anatomical and functional characteristics of this syndrome. Additionally, interventions combining tSCS with structured physical therapy (PT) programs have demonstrated synergistic effects, particularly when paired with task-oriented upper-limb training [12,14,16] or gait rehabilitation [10,13,15]. Despite the inclusion of AIS-D participants across studies, it remains uncertain whether individuals with CCS have been specifically represented. This knowledge gap reinforces the importance of characterizing injury syndromes and the motor and sensory evolution of both upper and lower limbs, as well as gait progression, in response to combined tSCS and rehabilitation protocols, critical outcomes addressed in the present report.

## 2. Materials and Methods

This case report describes a 20-year-old male diagnosed with CCS at the C7 level following a motor vehicle accident in February 2024. As a result of the injury, a cervical interbody cage was placed, and a predominant distal motor impairment in both hands and feet resulted. At hospital discharge, the subject was classified as AIS-D. Post-discharge, the patient underwent therapy at a private rehabilitation facility focused on gait re-education and training of both fine and gross grasp functions. The participant reported no use of medication for spasticity, pain, sleep disturbances, or other conditions during this period. At this stage of recovery, the individual required the use of a cane for ambulation. Twelve months after the SCI, the subject was enrolled in the study “Effect of Transcutaneous Spinal Cord Stimulation and Functional Rehabilitation on Motor Control in Individuals with Paraplegia due to Spinal Cord Injury”, approved by the Ethics Committee of Universidad Anáhuac México (approval number 202307; approval date: 23 August 2023). After providing informed consent, the participant initiated a 12-week intervention consisting of two sessions per week, combining tSCS with PT (tSCS+PT). Given that the participant identified improvement in gait as a priority due to mobility-related needs, the placement of the tSCS electrodes at the lumbar enlargement was selected in order to promote motor activation of the lower limbs [20,21]. The tSCS was delivered using two 2.5 cm round electrodes placed at the T11-12 and T12–L1 levels (cathodes) and 4 cm × 8 cm rectangular electrodes placed over the iliac crests (anodes). Stimulation parameters included a biphasic current with a pulse width of 280 μs per phase and a frequency of 30 Hz, delivered via a DS8R constant-current stimulator (Digitimer., Ltd., Letchworth Garden City, UK) controlled through LabChart Pro version 8 software (AdInstruments., Ltd., Dunedin, New Zeland). Stimulation parameters were selected based on previous research [22,23,24]. The stimulation intensity was increased in steps of 0.5 mA from 0 mA at 0.2 Hz and recorded in lower-limb muscles bilaterally, including rectus femoris (RF), hamstrings (HM), tibialis anterior (TA), and lateral gastrocnemius (LG). The stimulus intensity that produced Spinal Cord Motor Evoked Potentials (SCMEPs) in any muscle was subtracted by 10% and used as the reference tSCS current for the rest of the protocol (subthreshold intensity). The average applied current was 40 mA. In case of abdominal contractions or discomfort for the participant, the current was decreased. Impedance was monitored during sessions and kept at >2 kHz. During tSCS, the participant underwent PT for both the upper and lower limbs in sessions of approximately 40 min, including treadmill walking as a strategy to achieve the individual’s primary goal.

Outcome measures were collected at baseline and at the end of the 12-week intervention. Assessments included the following scales and tests: AIS, Penn, and Spasm Frequency Scale (PSFS), Modified Ashworth Scale (MAS), and handgrip strength (J00105 Hand Dynamometer Lafayette Instrument., Co., Layayette, IN, USA). Fine-motor performance was evaluated with the 9-Hole Peg Test (9HPT) and the Box and Block Test (BBT). Gait assessments included the 6-Minute Walk Test (6MWT) and the 3-Meter Walk Test (3MWT). Surface electromyography (sEMG) recordings were digitized at 2 kHz (bandpass 20–500 Hz, PowerLab., AdInstruments., Ltd., Dunedin, New Zeland) and stored for offline analysis. The same muscles used for the SCMEPs procedure were monitored during treadmill stepping at 3.2 km/h. sEMG analysis included the root mean square (RMS) and the area under the curve (AUC). For the RMS analysis, the gait episode was segmented into cycles using the RF as the reference muscle, and the step duration was obtained. The RMS was calculated during steeping for each burst of each muscle, and the mean and standard deviation values were calculated. A total of 11 cycles were analyzed at baseline and at 12 weeks. The AUC was obtained from the total duration of the gait trial at both time points, yielding one value per muscle. All analyses were performed in MATLAB version R2023b using custom-made scripts.

## 3. Results

**Figure 1:**
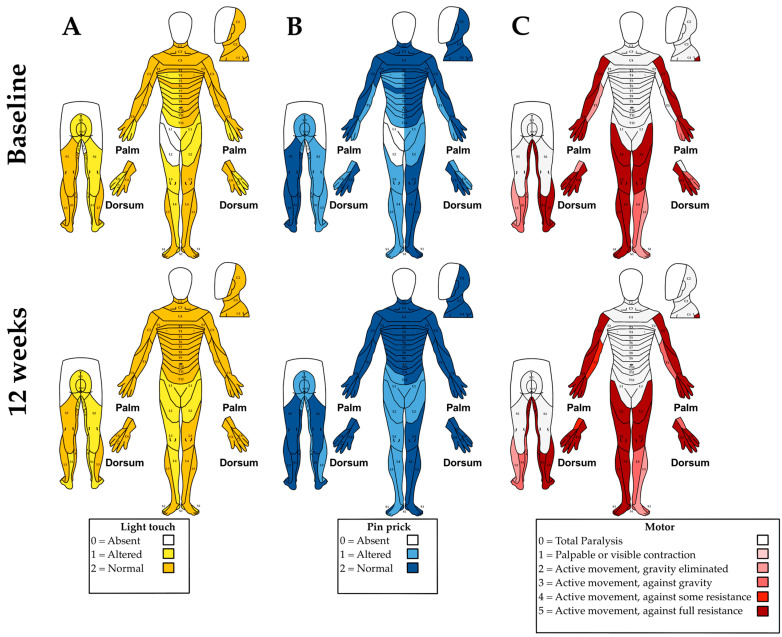
AIS evaluation at baseline (upper panels) and 12 weeks (lower panels). Sensory scores: (**A**) light touch and (**B**) pinprick; (**C**) motor scores.

Following 12 weeks of combined tSCS+PT, several clinically relevant changes were observed when compared with baseline assessments. The AIS-ASIA upper extremity motor score (UEMS) improved from 40 to 47 pts, while light touch sensation increased from 86 to 100 pts, and pinprick sensation from 85 to 98 pts. The lower extremity motor score (LEMS) remained unchanged at 43 pts. These findings are summarized in Figure 1.

Regarding the PSFS, spasm frequency decreased from 4 (spontaneous spasms occurring more than 10 times per hour) to 1 (spasms induced only by tactile stimulation). Severity, interference with function, and the presence of painful spasms remained at 0 at both time points. Clonus persisted at a score of 2 (sustained), and the plantar stimulation response remained at 1 (slight movement/extensor response). Deep tendon reflexes decreased from 2 (average/normal) to 1 (slightly decreased). These findings are detailed in Table 1. Spasticity was present only in the hands, with the left hand more affected. According to the MAS, the following improvements were observed: in the left hand, finger extension decreased from 1 to 0; thumb flexion decreased from 4 to 1. In the right hand, finger flexion and finger extension decreased from 1 to 0. The total MAS sum score for each hand is reported in Table 1.

The upper extremity motor function showed meaningful changes. In the BBT, performance improved from baseline (left hand: 26 blocks/min; right hand: 30 blocks/43 s) to 12 weeks (left hand: 30 blocks/min; right hand: 30 blocks/40 s). In the 9HPT, completion time improved from baseline (left hand: 2.43 min; right hand: 20 s) to 12 weeks (left hand: 1.28 min; right hand: 17 s). The left hand demonstrated the greatest improvement, reducing completion time by 1 min 15 s. Grip strength increased by 2 kg in the right hand (from 32 kg to 34 kg), with no change in the left hand (8 kg). Upper extremity evaluations are shown in Table 1.

Gait outcomes also improved. On the 6MWT, the patient increased the distance walked by 70.8 m (baseline: 224.4 m; 12 weeks: 295.2 m). In the 3MWT, the execution time decreased by 1.32 s (baseline: 7.96 s; 12 weeks: 6.64 s). These results are summarized in Table 1.

**Figure 2:**
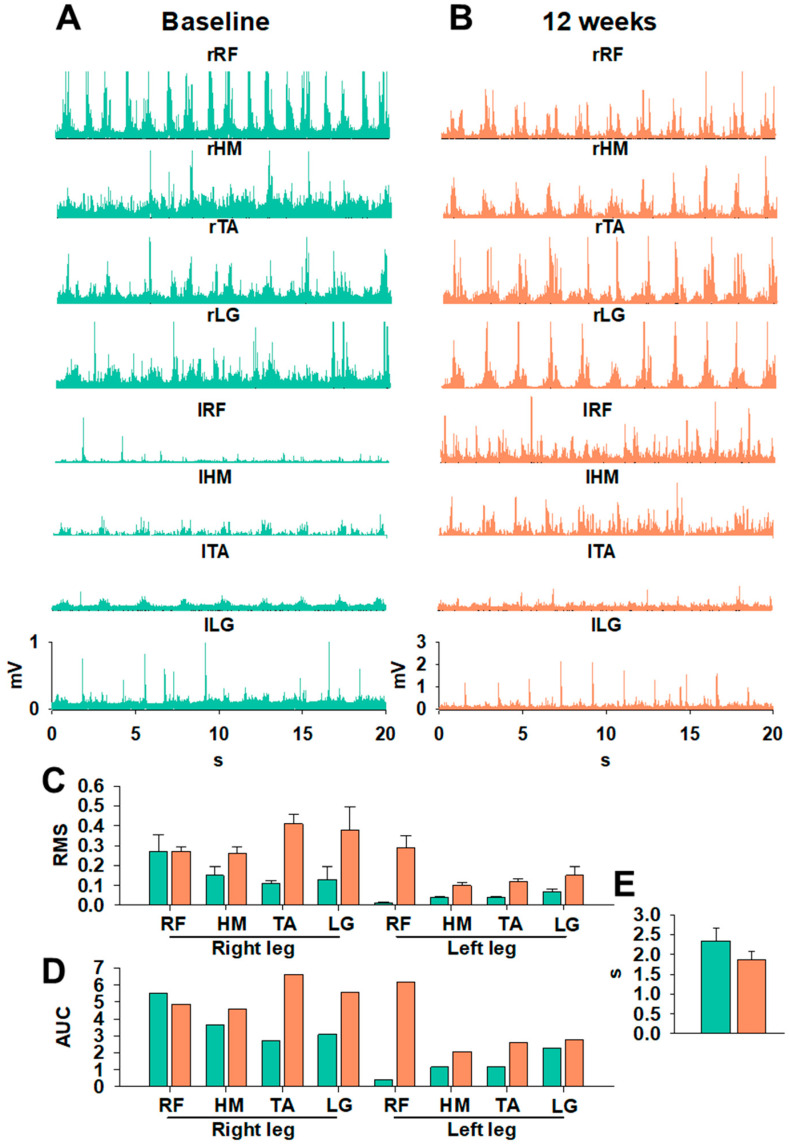
Stepping in treadmill (3.2 km/h) at (**A**) baseline and after (**B**) 12 weeks of tSCS+PT. Stepping trials in (**A**,**B**) were analyzed to obtain (**C**) RMS, (**D**) AUC, and (**E**) step cycle duration. Green, baseline; orange, after 12 weeks. Abbreviations: rRF (right rectus femoris), rHM (right hamstrings), rTA (right tibialis anterior), rLG (right lateral gastrocnemius), lRF (left rectus femoris), lHM (left hamstrings), lTA (left tibialis anterior), and lLG (left lateral gastrocnemius).

sEMG analysis revealed significant changes in neuromuscular organization during gait at baseline vs. 12 weeks, as shown in Figure 2A and 2B, respectively. Subjectively, prior to the intervention (Figure 2A), sEMG signals were irregular, with poorly defined peaks and limited differentiation between gait-cycle phase patterns typically associated with CCS involvement and impaired intermuscular coordination [25]. After tSCS+PT (Figure 2B), most of the lower-limb muscle groups demonstrated increased amplitude, improved peak definition, and enhanced rhythmic organization. The quantitative analysis of stepping in Figure 2C–E shows substantial neuromuscular improvements following 12 weeks of tSCS+PT, as reflected by changes in RMS, AUC, and step cycle duration (Figure 2C–E). In the right leg, RMS values increased at 12 weeks across all recorded muscles, including RF, HM, TA, and LG, indicating enhanced motor unit recruitment and a more robust activation profile during stepping. Similar but smaller magnitude changes were observed in the left leg, where RMS amplitudes also increased at 12 weeks, although baseline values were lower, particularly for RF, suggesting initial asymmetry consistent with CCS involvement (Figure 2C). The AUC analysis further highlights these improvements: all muscles in both legs showed increased AUC values after 12 weeks (Figure 2D), reflecting not only higher peak activation but also prolonged, more integrated muscle engagement across the stepping cycle. TA and LG showed particularly marked bilateral increases, supporting the interpretation of enhanced distal motor control and suggesting improved contributions to swing-phase dorsiflexion and stance-phase plantarflexion, respectively. In parallel, a decrease in cycle duration from baseline to 12 weeks was found (Figure 2E). The above findings are showcased in Supplementary Video S1 (Video S1). The speed of the treadmill was set at 3.2 km/h (≈2 mph). After 12 weeks, gait improvements suggest a more efficient execution of stepping, likely due to improved intermuscular coordination and reduced delays between activation bursts. Collectively, these findings corroborate the sEMG improvements and demonstrate that tSCS+PT facilitated locomotor output, reflected in higher amplitude, temporal integration, and rhythm of neuromuscular activity. Supplementary Video S1 shows improvements in gait after 12 weeks of tSCS+PT compared to baseline.

## 4. Discussion

This case report describes a 12-week program combining tSCS+PT applied at the thoracolumbar level (T11-L1) that produced meaningful improvements in motor, sensory, and functional performance in an individual with CCS. Prior to recruitment, the participant was undergoing conventional PT similar to that used in combination with tSCS in this study, suggesting that the changes reported in this case are attributable to the effects of noninvasive neuromodulation. The placement of the stimulation electrodes (thoracolumbar) was decided jointly with the participant, as his priority was to improve gait in order to facilitate mobility and activities of daily living.

The gains in upper-limb motor strength, light touch and pinprick sensation, hand dexterity, gait endurance, and neuromuscular coordination suggest that tSCS may effectively engage propriospinal and descending pathways that remain partially functional in CCS. These findings are consistent with recent evidence indicating that tSCS enhances spinal excitability and facilitates reactivation of dormant neural circuits in motor-incomplete SCI populations [10,12,13,14,15,16]. Improvements in upper-extremity function have been documented, particularly when cervical stimulation facilitates dorsal root afferent recruitment, thereby enhancing segmental and multisegmental integration [12]. Mild spasticity was limited to the hands, particularly the left hand (non-dominant), as shown in Table 1. The reduction in spasm frequency and improvements in spasticity are also consistent with emerging data showing that tSCS enhances both pre- and postsynaptic inhibitory control, modulating hyperexcitable reflex arcs commonly observed in incomplete SCI [26].

Interestingly, our results support the finding that neuromodulation applied at T11-L1 could impact cervical spinal circuits involved in upper-limb function. In this context, the motor and sensory improvements observed in this case study align with previous reports demonstrating that tSCS can modulate ascending inputs and enhance motor and somatosensory processing [13,15,16]. In the study by Inanici et al. (2021), two participants classified as AIS D were identified as having central cord syndrome (CCS); however, the effects of tSCS were evaluated exclusively in the upper limbs [16]. In this regard, our study provides additional evidence of functional improvements in both upper and lower limbs following the combined application of tSCS and physical therapy.

Furthermore, previous research has suggested that rehabilitation strategies simultaneously targeting both upper and lower limbs may promote thoracolumbar coupling and improve gait [27] through mechanisms that could involve the restoration of spinal reflexes [28]. In addition, Barss et al. reported that tSCS applied at cervical segments can modulate the activity of lumbar spinal circuits [29]. Although this latter study was conducted in a cohort of uninjured individuals, these findings may be extendable to populations with SCI. While further investigation is clearly required, it is plausible, albeit currently speculative, that a reciprocal effect on cervical spinal circuits may occur when tSCS is applied at the thoracolumbar level, which could at least partially account for our observed results.

Future studies using larger samples should systematically examine whether thoracolumbar stimulation affects upper-limb function, particularly in cervical injuries common to different spinal cord injury syndromes [4]. In this context, thoracolumbar rather than cervical stimulation may be preferable in an initial phase for patients with less severe cervical injuries who present sphincter control dysfunction [11], as improvements in this domain significantly enhance quality of life, facilitate physical activity, and increase patient confidence.

Gait performance notably improved, as reflected by increased distance on the 6MWT and reduced completion time on the 3MWT. These functional gains are supported by sEMG lower-limb recordings demonstrating increased amplitude, better-defined muscle activation peaks, and improved rhythmicity during the gait cycle (Figure 2), a pattern consistent with the reinforcement of central pattern generator activity described in recent locomotor neuromodulation studies with tSCS [13,15,30]. Distal musculature, particularly the tibialis anterior and gastrocnemius, showed the most pronounced improvements (Figure 2C,D), consistent with prior work showing that tSCS can enhance swing-phase dorsiflexion and push-off mechanics [15]. Overall, the pre-intervention gait cycle was characterized by rhythmic irregularity, poor limb alternation, and non-functional coactivation. Post-intervention recordings suggest more organized cycles, consistent activation peaks, and improved intermuscular coordination (Figure 2), suggesting functional modulation of spinal locomotor circuits. These neuromuscular changes align with the expected preservation of locomotor pathways and spinal plasticity in CCS. At the end of the intervention, the participant reported subjective improvements in finger control and gait, leading to discontinuation of cane use during ambulation at home.

Collectively, our results indicate functionally relevant reorganization of rhythmic and sequential neuromuscular patterns, suggesting that tSCS may facilitate enhanced modulation of spinal circuits to improve gait expression in individuals with CCS. Importantly, the neuromuscular reorganization observed post-intervention suggests that, even in CCS, where disproportionate upper-limb impairment is typical, spinal networks retain the capacity for adaptive plasticity when appropriately stimulated. This is consistent with previous evidence of functional recovery beyond expected natural plateaus when neuromodulation is integrated with task-specific training [12,13,14,15]. However, this study has limitations inherent to a single-case design. The absence of a control condition limits causal inference, and the stimulation parameter for this participant may not generalize to all individuals with CCS. Additionally, while no adverse events occurred, recent studies suggest that tSCS may modulate autonomic circuits in variable ways, underscoring the need for careful monitoring beyond sensory and motor function in future work [11,31]. In this case report, priority was given to the evaluation of changes in sensorimotor function, as the participant did not present signs or symptoms of dysautonomia. Moreover, the baseline assessment using the Autonomic Standard Assessment Form did not identify dysfunction in any autonomic system, such as cardiovascular, respiratory, or sacral function. However, a recent report described that in two individuals with cardiovascular autonomic dysfunction, tSCS may exacerbate abnormal inhibitory responses of the sympathetic nervous system [31]. In light of this evidence, we consider that careful characterization of autonomic function in individuals receiving tSCS is warranted in future studies.

Moreover, the vast majority of current evidence does not allow a definitive determination of whether individuals with CCS have been included in tSCS studies. The broad eligibility criteria, particularly those requiring incomplete cervical injuries and preserved ambulation, align with the clinical features of CCS, suggesting that some participants may have been included unintentionally. This gap is significant, as CCS represents a considerable proportion of cervical spinal cord injuries [4] and may exhibit distinct responses to neuromodulation due to its disproportionate impact on upper versus lower extremity function.

## 5. Conclusions

This case report shows that a 12-week program combining tSCS and PT can enhance motor, sensory, and functional performance in the upper and lower limbs in a young adult with CCS. Thoracolumbar electrical stimulation produced meaningful improvements in gait endurance and neuromuscular coordination in the lower limbs, as revealed by sEMG, with more organized, phase-appropriate activation patterns following the intervention. These findings were accompanied by reductions in spasticity and spasm frequency. Importantly, improvements also positively impacted upper-limb strength, fine motor control, and sensory scores. Overall, our results suggest a favorable neuromodulation effect on spinal circuitry and residual descending pathways.

These findings provide preliminary evidence that tSCS may serve as a valuable adjunct to conventional rehabilitation for individuals with mild, incomplete spinal cord injuries, including the CCS population, for whom targeted neuromodulation has not been explicitly reported. The absence of adverse effects further supports the safety and feasibility of integrating tSCS into multidisciplinary rehabilitation programs.

## Figures and Tables

**Table 1:** Clinical and functional outcome measures at baseline and after 12 weeks of tSCS+PT.

Scale/Items	Baseline	12 Weeks
Penn Scale		
Frequency of spasms	4	1
Severity	0	0
Interference with function	0	0
Painful spasms	0	0
Clonus score	2	2
Deep tendon reflex	2	1
Response to plantar stimulation	1	1
Ashworth Modified Scale		
Right hand	2	0
Left hand	5	1
Box and Block Test		
Right hand	30 in 43 s	30 in 40 s
Left hand	26 in 1 min	30 in 1 min
9-Hole Peg Test		
Right hand	20 s	17 s
Left hand	2.43 min	1.28 min
Stepping test		
6-Minute walk test (min)	224.2	295.2
3-Meter walk test (s) ^1^	7.96 ± 0.2887	6.64 ± 0.1375

^1^ Three attempts were performed.

## Data Availability

Data are available from the corresponding author upon request. The data that support the findings of this study are not publicly available due to the sensitive and identifying nature of the data.

## Supplementary Materials

The following supporting information can be downloaded at: https://www.mdpi.com/article/10.3390/neurolint18020031/s1, Video S1: Stepping on a treadmill.

## Abbreviations

The following abbreviations are used in this manuscript:

CCS	Central Cord Syndrome
tSCS	Transcutaneous Spinal Cord Stimulation
SCI	Spinal Cord Injury
PT	Physical Therapy
AIS	American Spinal Injury Association Impairment Scale
ASIA	American Spinal Injury Association
MAS	Modified Ashworth Scale
PSFS	Penn and Spasm Frequency Scale
3MWT	Three-Meter Walk Test
6MWT	Six-Minute Walk Test
9HPT	Nine-Hole Peg Test
BBT	Box and Block Test
EMG	Electromyography
RF	Rectus Femoris
HM	Hamstrings
TA	Tibialis Anterior
LG	Lateral Gastrocnemius
SCMEPs	Spinal Cord Motor Evoked Potentials
sEMG	Surface Electromyography
RMS	Root Mean Square
AUC	Area Under the Curve
UEMS	Upper-Extremity Motor Score
LEMS	Lower-Extremity Motor Score
